# Rational design of arbitrary topology in three-dimensional space *via* inverse calculation of phase modulation

**DOI:** 10.1515/nanoph-2024-0001

**Published:** 2024-02-15

**Authors:** Hwanseok Chang, Sungjoo Kwon, Gwangmin Bae, Seokwoo Jeon

**Affiliations:** Department of Materials Science and Engineering, Korea University, Seoul 02841, Republic of Korea

**Keywords:** inverse calculation, proximity-field nanopatterning, adjoint method, particle swarm optimization

## Abstract

Recent advances in nanotechnology have led to the emergence of metamaterials with unprecedented properties through precisely controlled topologies. To explore metamaterials with nanoscale topologies, interest in three-dimensional nanofabrication methods has grown and led to rapid production of target nanostructures over large areas. Additionally, inverse design methods have revolutionized materials science, enabling the optimization of microstructures and topologies to achieve the desired properties without extensive experimental cycles. This review highlights the recent progress in inverse design methods applied in proximity-field nanopatterning. It introduces novel approaches, such as adjoint methods and particle swarm optimization, to achieve target topologies and high-resolution nanostructures. Furthermore, machine learning algorithms for inverse design are explored, demonstrating the potential efficacy of the phase-mask design. This comprehensive review offers insights into the progress of inverse design using phase modulation to realize target topologies of nanostructures.

## 1 Introduction

Recent advances in nanotechnology have ushered in a new era of engineering. For instance, metamaterials designed to exhibit unique properties that are not based on their chemical composition or atomic bonding but on precisely controlled nanoscale structural arrangements have emerged with the development of nanotechnology. These new materials have demonstrated unprecedented optical [1], mechanical [2], [3], [4], and thermal properties [5], [6], which cannot be obtained using conventional thermodynamic processing of materials [7]. With the exploration of materials for nanoscale technology, interest in three-dimensional (3D) nanofabrication technologies that enable rapid and large-scale production of rationally designed nanostructures [8], [9], [10] has surged.

**Figure 1:**
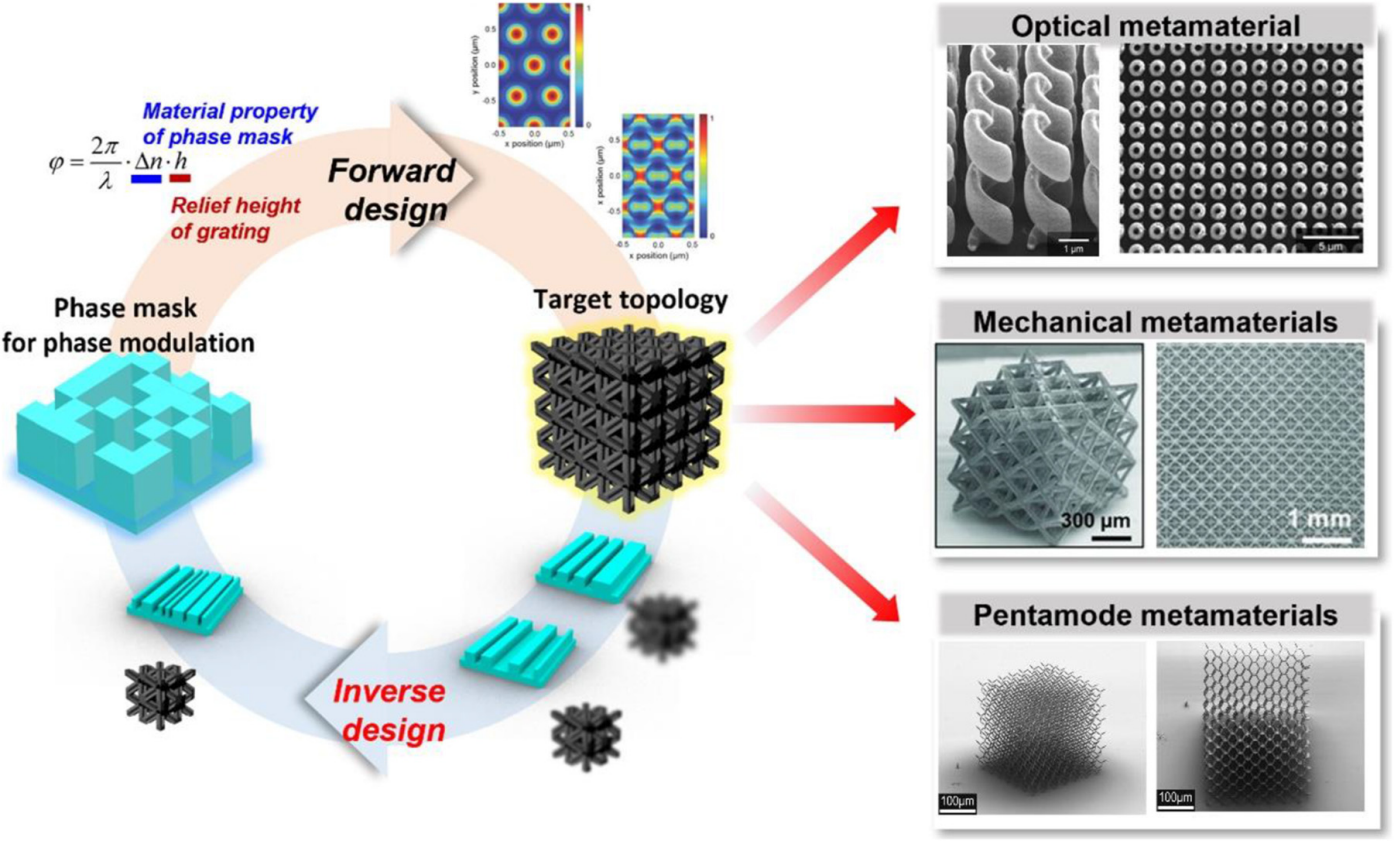
Schematic of forward and inverse design directions for fabricating target topology using PnP (reproduced from [14], [15], [16]).

Furthermore, owing to the exponential growth in computing power and emergence of innovative computational methods, the field of materials science has extended its methodology by virtue of the inverse calculation process [11], [12], [13]. While the rational design and production of new materials should have been preceded by analyzing the material’s properties in conventional research, inverse calculation and AI methodologies enable the exploration of optimized microstructures, constituents, or topologies of materials to achieve the desired material properties without repetitive and costly experimental cycles (Figure 1) [14], [15], [16]. For example, Ha et al. scrutinized an inverse design methodology based on machine learning and developed rapid inverse design tools to realize an optimized architectural topology that ensures arbitrary mechanical behaviors. To date, the successful fabrication of optimized microstructures has been reported using additive manufacturing, including two-photon lithography [17], [18], [19], direct laser writing [20], and laser powder bed fusion [21], [22], etc. While these 3D printing techniques can be versatile for producing arbitrary topologies derived by inverse calculation, the direct printing of topologies with smaller submicron feature sizes requires tremendous time and cost to produce at industrial scales. Research on the methodologies for producing target topologies derived from a wide variety of applications in a large area remains challenging.

**Figure 2:**
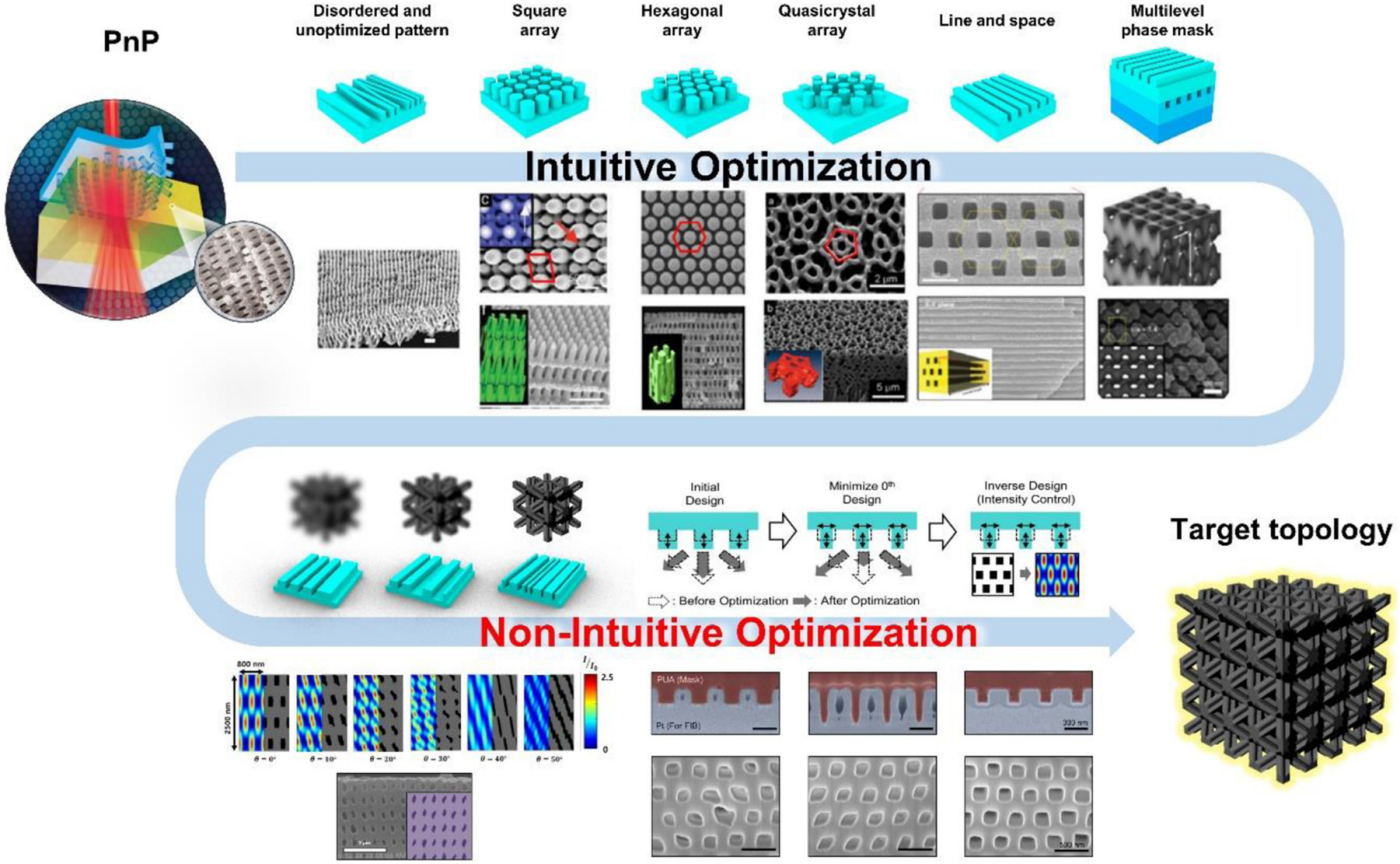
Illustration of the research progress and optimization strategies of PnP (reproduced from [9], [23], [24], [26], [28], [29], [30], [39], [40]).

Among the various fabrication methods, proximity-field nanopatterning (PnP) has been regarded as a promising technology for realizing 3D nanostructures over a large area using the interference and diffraction of light sources [9], [10], [23], [24], [25], [26]. The PnP process generates 3D diffraction patterns from the collimated light that illuminates the area in direct contact with the phase mask and directly transfers it to the photopolymer. Owing to the use of optics in conformal contacts, this method is highly stable and reproducible with negligible sensitivity to ambient vibrations and external conditions [24]. To date, several periodic topologies including body-centered tetragonal (BCT) [9], hexagonal close-packed (HCP) [26], [27], quasicrystal [23], [28], high aspect ratio [26] long nanochannel arrays [29], and woodpile symmetry [30] have been fabricated and applied to photonic crystals [30], energy devices [31], [32], [33], sensors [34], [35], and structural materials [36], [37], [38]. However, these topologies can be simply predicted by basic optics theories. When producing more complex and high-contrast nanostructures, it could be much more difficult to realize using conventional methodology. Further investigation to realize target topologies using PnPs will expand the scope of applications; however, it is still in its infancy. In this review, we introduce recent advances in inverse design methods applied to PnPs. In contrast to conventional research, inverse design methodologies have enabled the effective and rapid identification of design parameters that were challenging to consider simultaneously using traditional computational methodologies (Figure 2). First, we introduce the adjoint method for the inverse design of PnP, thereby realizing an untouched lattice and nanotopology motif. Next, an innovative approach utilizing particle swarm optimization (PSO) is discussed to produce the high spatial resolution of 3D nanostructures. Finally, we present the inverse design of metasurfaces using a machine-learning algorithm, demonstrating the potential of machine learning as an effective tool for phase-mask design. Our review provides a comprehensive outlook and perspectives on the inverse design methodology of phase modulation to realize an arbitrary topology of nanostructures over a large area.

## 2 Conventional approach to optimize design parameter of PnP

**Figure 3:**
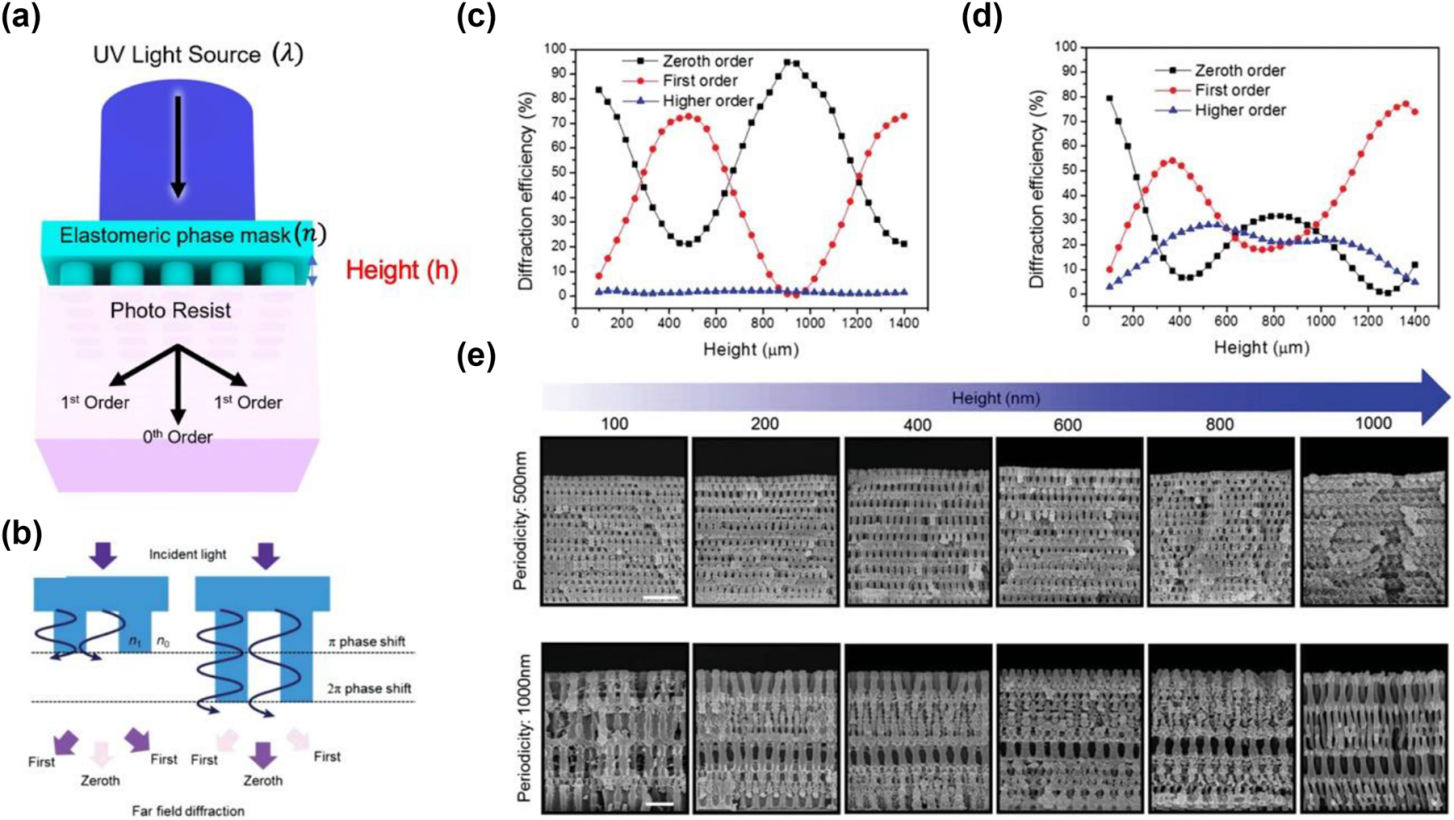
Conventional approach to optimize PnP process. (a) Basic optical components of PnP. (b) Schematic illustrations of simple binary grating showing the optimized condition satisfying *π* phase shift. Computed diffraction efficiencies of diffraction orders as functions of the height of a nanograting with (c) 500 nm and (d) 1000 nm periodicity. (e) SEM images of 3D nanostructures obtained by PnP by modulating the height of a nanograting ((b)–(e) reproduced from [26]).

PnPs have been used to produce periodic 3D nanostructures with a wide variety of scales and symmetries. The fundamental principles of PnP depend on an optical phase mask consisting of periodic relief structures [24], [26], [29]. The binary grating of the phase mask enables the generation of 3D diffraction patterns in the near field, directly imprinting the photopolymers. Hyun et al. reported intuitive control of diffraction from a phase-mask grating to investigate the optimized topology for the highest spatial resolution [27]. In terms of basic scalar theory, it is well-known that high spatial resolution can be achieved when the phase difference between periodical grating and surround media is *π*, *i.e.* for a *π* phase shift condition, as well as the lowest zeroth-order efficiency. The phase shift (*φ*) of grating materials can be formulated by [27], [29]

(1) $$\begin{array}{c} \varphi =\frac{2\pi }{\lambda }\Delta nh \end{array}$$

where *λ* is wavelength of incident light source, Δ*n* is difference of refractive index between nanograting and the other medium (air), and *h* is height of the relief structures (Figure 3a). Furthermore, when the periodicity (*p*) of the grating is similar to the wavelength of the light source, the actual variation of the zeroth order efficiencies deviates unexpectedly concerning the periodicity and relief height. Hence, Klein’s parameter (*Q*) should be considered as another parameter that determines the resolution of nanostructures [41].

(2) $$\begin{array}{c} Q=\frac{2\pi h\lambda }{p^{2}} \end{array}$$

The authors assumed the simple physical grating model, for which fill factor is fixed by 50 % [42], [43]. Under these conditions, the primary consideration for the phase shift is the relief height (*h*). Thus, the spatial resolution of 3D nanostructures can be tailored in a straightforward manner by modifying *h*. They revealed that well-defined 3D nanopatterns with a phase mask are realized by odd multiples of the *π* phase shift conditions (Figure 3b). They scrutinized the calculated diffraction efficiencies as a function of the grating relief height with representative periodicities of 500 nm (Figure 3c) and 1000 nm (Figure 3d), and the resultant comparison between the diffraction efficiencies and 3D nanostructures is summarized in Figure 3e. Most of the graphs tended to show simple sinusoidal and periodic variations, and the diffraction efficiencies of the phase mask with a larger periodicity (1000 nm) exhibited a more complicated trend owing to the larger number of diffraction orders. The resultant scanning electron microscopy (SEM) images of the 3D nanostructure produced by PnP showed good agreement with the assumption that the geometry of the phase mask with minimal zeroth-order diffraction efficiency exhibited the highest spatial resolution. While this straightforward approach provides intuitive solutions for achieving high-contrast 3D nanopatterns given the periodicity, refractive index, and fill factor, it only considered a single independent variable (*h*) to characterize the phase-shift condition. Consequently, it has limitations to comprehensive optimization considering other variables determining the phase shift condition, such as *λ*, Δ*n*, and fill factor, at the same time. Thus, non-intuitive methodologies that provide a more comprehensive consideration of the parameters of the PnP and realize the optimized topology and resolution of 3D nanostructures should be considered.

## 3 Inverse design in PnP with adjoint-based method

In this section, we introduce recent efforts to apply inverse calculations to the PnP process. Although the traditional intuitive method has proven its usefulness in predicting and producing periodic 3D topologies [9], [10], [23], [24], [25], [26], it encounters challenges when dealing with target topologies with high design freedom and spatial resolution, considering all process parameters. The conventional simple periodic phase mask used to generate 3D nanostructures only produces specific topologies under a vertical light source. To expand the range of 3D nanostructures, more complex and nontrivial grating patterns of the phase mask, which have a high degree of freedom, must be considered. However, exploring the design space of phase masks using conventional approaches such as genetic algorithms (GA) pose a significant challenge in terms of time and cost.

Recently, advances in computing power and methods have led to the realization of an optical design that provides a larger design space compared to traditional methods [44], [45]. Especially, in the field of photonic devices such as optoelectronics [46], metasurfaces [47], [48], [49], and metalens [50], [51], [52], the emergence of complex structures through inverse design has enabled sophisticated control over the phase, amplitude, and polarization of light, leading to the possibility of achieving extraordinary optical properties. Among the various inverse calculation methods, the adjoint method, which is based on physical gradients, is a versatile technique that can quickly compute the gradients of the figure of merit (FoM) for a large number of design variables. It generates a desired target hologram through a direct computational path and reciprocity relationship between forward and adjoint response functions [53], [54], [55]. Nam et al. proposed a nonintuitive design system to diversify the lattice and motifs of 3D nanostructures using a novel nanograting phase mask [39]. They initially adjusted the angle of the incident light source to obtain a rectangular 2D lattice. Unlike the existing hexagonal lattice typically produced by conventional PnPs, the rectangular lattice plays a crucial role in determining the yield in semiconductor processes. Nevertheless, the implementation of a rectangular array of lattices using an existing vertically incident beam was initially considered impossible.

**Figure 4:**
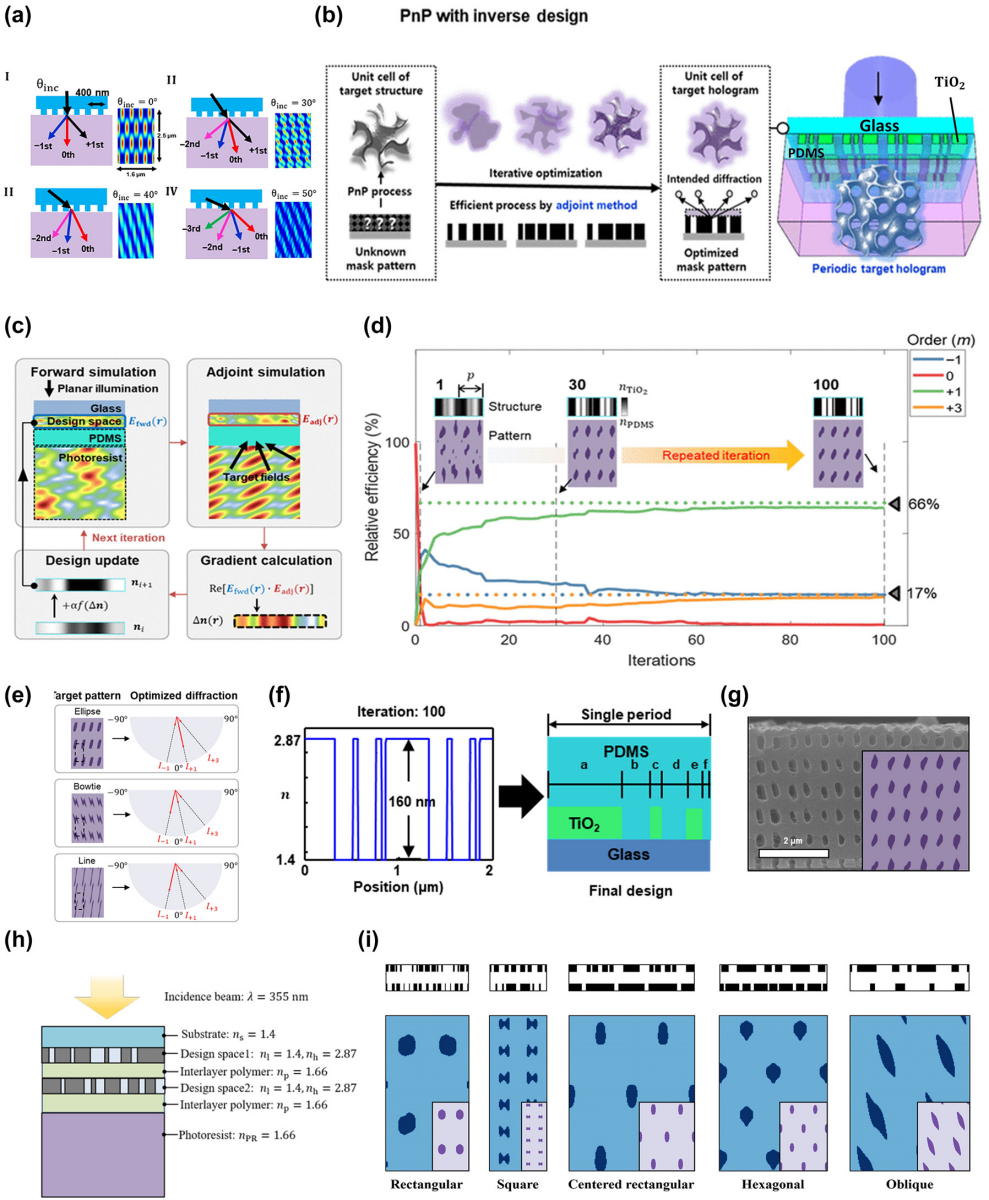
Inverse design of PnP process by adjoint method. (a) Diffraction orders and patterns induced by 1D grating phase mask as a function of incident angles of the light source. (b) Schematic illustration of gradient-based optimization with the adjoint method. (c) One iteration process of the adjoint method. (d) Relative efficiencies of diffraction orders as a function of iterations, showing that rectangular lattices are produced after 100 iteration processes. (e) Manipulation of the lattice and motif by selecting and adjusting the intensity of each diffraction orders. (f) Resultant geometry of the phase mask derived by adjoint method, consisting of TiO_2_ and PDMS. (g) SEM cross-sectional images of nanochannels fabricated via phase masks produced by inverse designing. (h) Simulation setting for inverse design of bilayer phase mask. (i) Optimized bi-layer phase mask with resulting patterns (blue) and target patterns (purple) of all 2D Bravais lattices ((a)–(g) reproduced from [39], (h)–(i) reproduced from [57]).

The angle of the incident beam on the periodic phase mask determined the number of diffraction orders and generated four different stages based on the relationship between the grating vector of the phase mask and the horizontal component of the wave vector (Figure 4a). However, when considering the modification of the light angle, it is difficult to simultaneously control the diffraction angles and efficiencies, preventing the independent tailoring of the lattice and motif in the topology. To overcome the limitations of the angle-resolved PnP process and enhance design flexibility, they applied the adjoint method to simultaneously realize the target motifs and lattices. Conventional numerical simulation techniques require discrete-time calculations to determine FoM. This involved *N* + 1 forward simulations, where *N* represents the number of design variables. By contrast, the adjoint method enables gradient computation with only two simulations, irrespective of the number of parameters. Forward calculation (*E*_fwd_(*r*)) and inverse calculation (*E*_adj_(*r*)) recorded the electric field within the phase-mask region. Although both the forward and inverse calculations utilize the same phase-mask configuration, they are stimulated by different sources. The forward calculation is excited by a normally incident plane wave, whereas the inverse calculation is encouraged by a time-reversed version of the target interference pattern. After reformulating the equations, the refractive index gradient of the FoM can be computed in the design space based on the interference between the forward and inverse fields [56]. These forward and inverse simulation steps were iteratively performed throughout the optimization process. In the iteration process, the refractive index for each pixel in the phase mask is considered as a continuous variable within the range of *n*_PDMS_ ≤ *n* ≤ $n_{{\text{TiO}}_{2}}$ . Starting with a random initial configuration, the composition of the phase mask was progressively updated in each iteration. Each iteration encompasses two simulations (forward and inverse): gradient computation and updates of design variables. The refractive index distribution of the phase mask is updated using a gradient-based optimization algorithm, such as the gradient ascent method or limited-memory Broyden–Fletcher–Goldfarb–Shanno algorithm (L-BFGS). After each iteration, slight adjustments were made to update the refractive index distribution of the phase mask, with the aim of enhancing its efficiency in terms of the FoM (Figure 4c).

Asymmetric scattering in the designed mask enables the production of an arbitrary lattice and a motif of nanopatterns by precisely controlling the design parameters, including the number, direction, amplitude, and phase of the multibeams. The spatial intensity profile of the multibeam interference can be formulated as [58]

(3) $$\begin{array}{c} I\left(r\right)=\overset{N}{\underset{m=1}{\sum }}{\left|E_{m}\right|}^{2}+\overset{N}{\underset{m\neq l}{\sum }}E_{m}^{*}\cdot E_{l}\exp\left[i\left(k_{m}-k_{l}\right)\cdot r\right] \end{array}$$

where *r* is the position vector and *E*_*m*_ and *k*_*m*_ are the complex amplitude and wave vector of the *m*-th beam, respectively. To generate a 2D pattern, the difference vectors *k*_*m*_ − *k*_*l*_ must lie in the same plane for all pairs of *m* and *l*. From a mathematical perspective, the formation of all 2D Bravais lattices requires only three beams in PnP [58]. For example, Figure 4d illustrates the evolution of the pattern and phase mask design during the optimization of a rectangular lattice topology with an elliptical motif through multiple iterations. A rectangular lattice can be fabricated using three beams that propagate at angles relative to the normal direction: −1st, 1st, and 3rd. By adjusting the relative intensities of the three beams, elliptical, bowtie, and line-segment motifs were obtained (Figure 4e). The FoM for realizing a rectangular array of ellipses is defined as

(4) $$\begin{array}{c} \text{FoM}=-\underset{m}{\sum }w_{m}\left|T_{m}^{\text{tgt}}-T_{m}^{\text{fwd}}\right| \end{array}$$

where $T_{m}^{\text{tgt}}$ and $T_{m}^{\text{fwd}}$ represent the actual and target relative amplitudes of the m-th beam, respectively, and the difference is multiplied by the weighting factor *w*_*m*_. Initially, a randomly generated interference pattern was inserted. In each iteration, one forward and one inverse simulation were performed as described earlier. Subsequently, the design variables were updated using the gradient vector calculated from the recorded forward and inverse fields. This process was repeated for 100 iterations, as shown in Figure 4d. After iterations, a nontrivial grating pattern was designed on a phase mask consisting of different refractive indices, including those of PDMS (*n*_PDMS_ = 1.40) and ${\text{TiO}}_{2}\left(n_{{\text{TiO}}_{2}}=2.87\right)$ (Figure 4f). The multilayer phase mask consists of nontrivial TiO_2_ patterns (thickness of 160 nm) and PDMS substrate with a period of 1 μm. A target structure similar to the simulated result was obtained using the designed phase mask (Figure 4g). Recently, it has been demonstrated that complete control over the amplitude, polarization, and phase of each diffraction beam can be achieved through a bi-layer metasurface [59]. Kim et al. attempted to enhance the design flexibility of PnP by designing a bi-layer phase mask composed of substrate (*n*_*s*_ = 1.40), PDMS (*n*_*l*_ = 1.40), TiO_2_ (*n*_*h*_ = 2.87), and interlayer polymer (*n*_*p*_ = 1.66) using wavelength 355 nm (Figure 4h) [57]. They also added an additional layer of the phase masks, expanding the range of the design parameters. Utilizing the adjoint method for faster mask optimization, they successfully reproduced all 2D Bravais lattices with various motifs through FDTD simulations, demonstrating the potential advancement of PnP (Figure 4i). This research offers a promising outlook for the rapid and precise fabrication of arbitrary structures previously considered unattainable.

## 4 PnP optimization with PSO algorithm for high-contrast nanopattern

**Figure 5:**
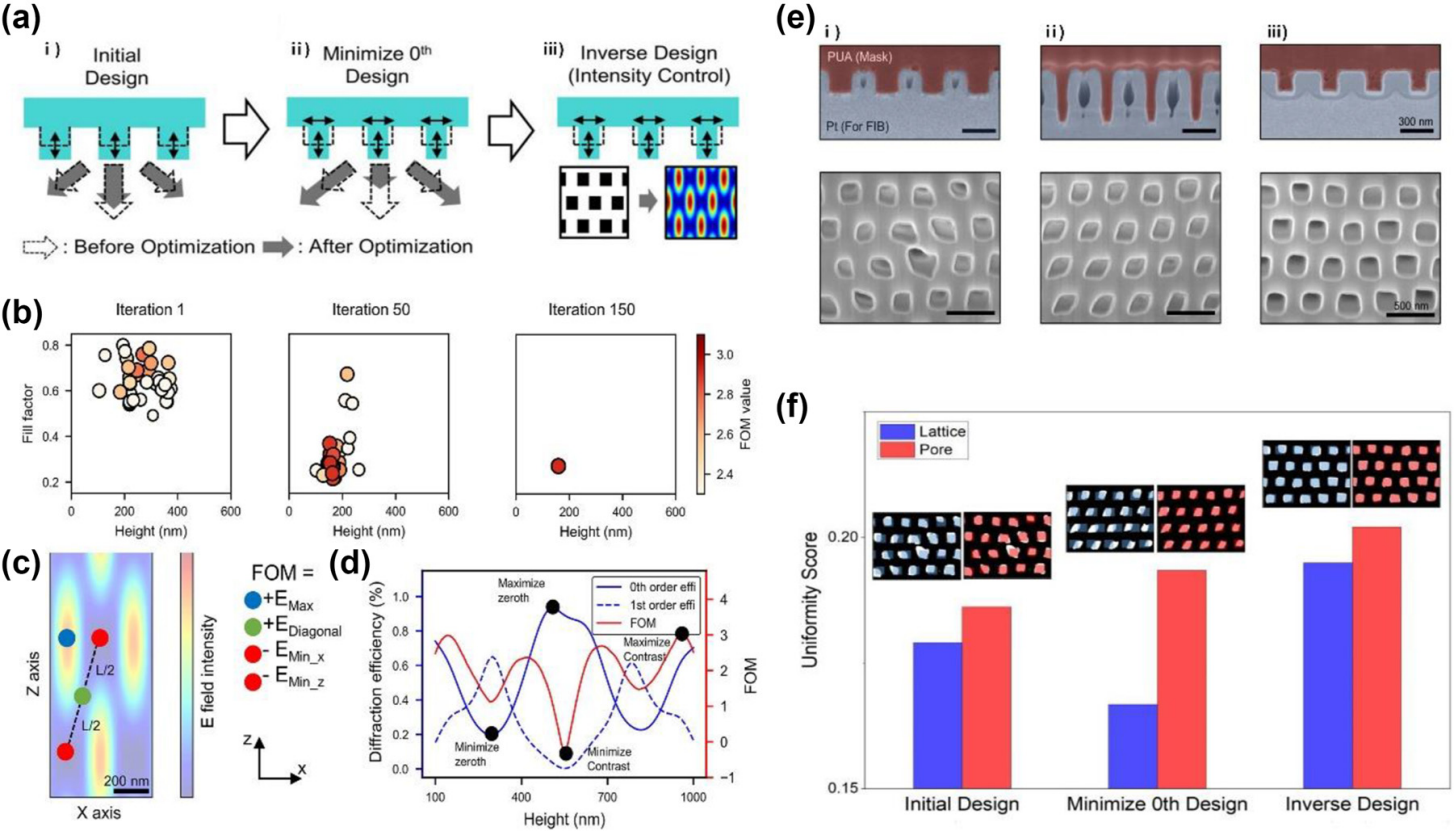
Inverse optimization of the PnP process by a PSO algorithm for high-contrast 3D nanopatterning. (a) Progress of optimizing the phase mask: (i) initial design based on fill factor of 0.5, (ii) minimize zeroth-order design (iii) inverse design method using FoM. (b) Convergence tendency of PSO algorithm, converging to the target solution as the iteration number increases. (c) Distribution of electric field intensity produced by PnP and intensity points of FoM. (d) Diffraction efficiencies and FoM of the phase mask as a function of relief height. The periodicity and fill factor were fixed by 400 nm and 50 %, respectively. (e) Produced phase mask geometries and resultant nanostructures of (i) initial design, (ii) minimizing zeroth-order design, and (iii) inverse design considering FoM. (f) Comparison of uniformity of lattices (blue) and pore morphologies (red) based on SSIM analysis (Inset: experimental lattices and pore morphologies matched with ideal hexagonal patterns) ((a)–(f) reproduced from [40]).

Unlike gradient-based models, several efforts have been made to utilize non-gradient-based global optimization methods, such as GA [60] and PSO algorithms [61] for the inverse design of target nanostructures beyond differentiable design fields. While genetic algorithms evolve a population of individuals using genetic operators such as crossover probability and mutation probability, PSO operates by having particles adjust their velocity and position exclusively to find optimal solutions [62]. Therefore, it is suitable as a fast problem solver of simplistic conditions that optimizes several parameters. Recently, Lee et al. applied the PSO algorithm to the PnP technique to obtain an optimized phase-mask geometry for high-contrast 3D nanostructures [40]. To prevent the derivation of local optimal data during the iteration processes, the authors customized a PSO algorithm for the PnP process by setting user-defined data ranges for each design parameter [63], [64] and considering the boundary conditions for the parameters to avoid phase-mask collapse [65]. They investigated three representative simulation models of high-contrast patterns (Figure 5a). First, the authors selected the initial design of the phase mask optimized using a conventional method in which only the height of the nanograting was considered. Furthermore, they attempted to determine the phase-mask geometry with the lowest zeroth-order efficiency by considering all components of the phase mask, including not only the height but also the fill factor (FF) and refractive index of the grating, referred to as (ii) minimizing the zeroth-order design. Finally, they proposed an FoM to directly reflect the electric field intensity distribution of a given 3D diffraction pattern, denoted as (iii) inverse design. After 150 iterations, the optimal geometries of the phase mask for the highest electric field contrast were derived (Figure 5b). The FoM of the BCT symmetry can be formulated as

(5) $$\begin{array}{c} \text{FoM}=E_{\max}^{2}+E_{\mathrm{diag}}^{2}-E_{\text{minx}}^{2}-E_{\text{minz}}^{2} \end{array}$$

where *E*_max_ is the maximum intensity, *E*_diag_is the intensity of the middle point of the two minimum points, *E*_minx_ is the intensity of the minimum point along the same *x*-axis from the maximum point, and *E*_minz_ is the intensity of the minimum point along the same *z*-axis from the maximum point (Figure 5c). To realize hexagonal interference patterns followed by the Talbot effect, it is crucial to establish the lowest intensity points in two regions (*E*_minx_ and *E*_minz_) and the highest intensity points in the other regions (*E*_max_ and *E*_diag_). Notably, the resulting geometries of the phase masks are entirely dissimilar from each other, and the simulated results between (ii) minimizing the zeroth-order design and (iii) the inverse design seem to be contradictory. Specifically, the diffraction efficiency of the zeroth order from (iii) the inverse design was higher than that from (ii) minimizing the zeroth-order design (Figure 5d). This implies the challenge of achieving high spatial resolution using phase-mask geometry derived from inverse design, particularly when considering the zeroth-order diffraction efficiency. However, the cross-sectional SEM images obtained from the three cases show that the phase mask acquired through the inverse design achieved the highest resolution and integrity of the nanopattern (Figure 5e).

To quantitatively analyze the structural integrity and uniformity, the Structural Similarity Index Measure (SSIM) model was utilized, which can evaluate the similarity between two images [66], [67]. The SSIM algorithm scores the uniformity of an input binary image by comparing an ideal hexagonal lattice with a converted binary image. Consequently, the 3D nanostructures obtained using the phase mask from the inverse design demonstrated the best uniformity compared with others (Figure 5f). The new type of inverse design system suggests optimal design parameters to produce 3D structures with the highest intensity contrast using PnP, considering all phase-mask geometry parameters. Although the phase mask design is currently limited by the 1D grating, their innovative approach, using a specific electric field intensity point as the FoM, can provide effective methods for expanding the range of topologies that have not been captured through conventional methods.

**Figure 6:**
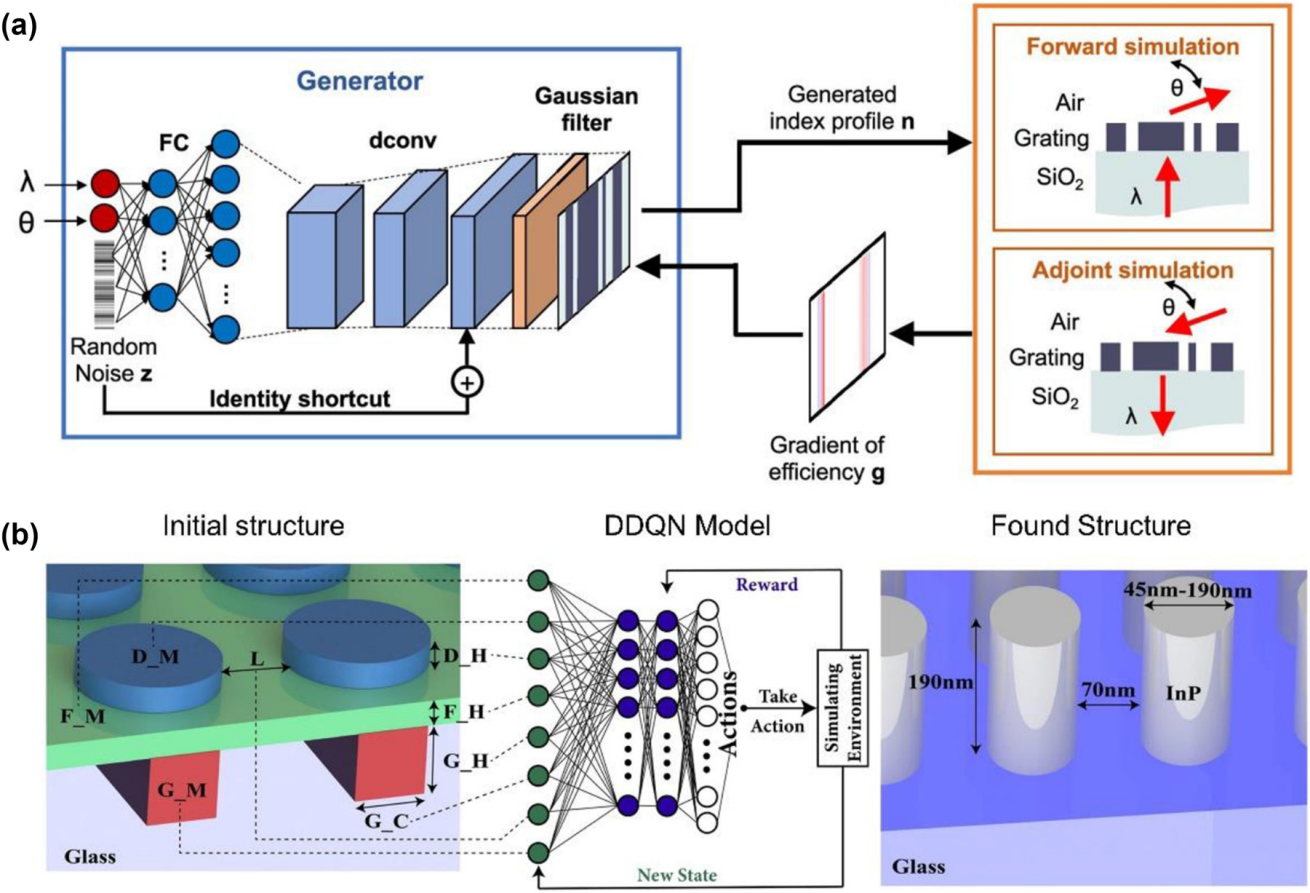
Application of machine learning frameworks in nanophotonic device to optimize topology. (a) Schematic of the global optimizer based on generative neural network for metasurface generation. In each training iteration, a batch of devices is generated, and efficiency gradients for each device are calculated through forward and adjoint electromagnetic simulations. These gradients are backpropagated through the network to adjust the weights of the neurons ((a) reproduced from [68]). (b) Schematic of application of DDQN algorithm for discovering high-efficiency holograms ((b) reproduced from [69]).

## 5 Inverse design for metasurface by machine learning

Metasurfaces are flat optical components composed of subwavelength-structured elements designed to exhibit specific electromagnetic responses [70], [71], [72]. Typically, designing metasurfaces to achieve the desired electromagnetic response involves numerous electromagnetic simulations. Machine learning is a promising candidate for reducing the computational cost and time required for inverse design of metasurface [73], [74], [75]. Given that the phase mask in PnP represents a type of dielectric metasurface utilized for interference and diffraction of the light source, exploring inverse calculations of metasurfaces could offer a promising research avenue for phase mask design.

Jiang et al. reported a global optimizer based on machine learning, specifically a generative neural network, that produced highly efficient topology-optimized metasurfaces (Figure 6a) [68]. This network initially generates a distribution of topologies that broadly sample the design space. Subsequently, it adjusts and improves this distribution, directing its focus towards favorable regions within the design space throughout the optimization process. This approach prevents the optimal topology from becoming stuck in a local minimum, mitigating the need to restart the optimization from the beginning (cold start) when using different initial parameters such as wavelengths or deflection angles. The adjustment of each neuron’s weight was achieved through back-propagation, utilizing the gradient computed with the adjoint method. Furthermore, greater focus was placed on high-efficiency devices by employing a weighted average proportional to the exponential efficiency. The adjustment of each neuron’s weight was achieved through back-propagation, utilizing the gradient calculated using the adjoint method. Furthermore, more emphasis was placed on high-efficiency topologies by employing a weighted average proportional to exponential efficiency. Through this approach, the neural network was able to learn the nonlinear relationship between the topology of the metasurface and the optical response. Furthermore, the generated metasurface exhibited a transmission efficiency approximately twice as high within the trained range of physical parameters compared to previous adjoint-based optimization results.

Additionally, Sajedian et al. utilized a machine-learning method called a double deep Q-learning network (DDQN) as an approach for the inverse design of metasurfaces (Figure 6b) [69], [76]. A DDQN is a reinforcement-learning technique in which an agent performs a task, receives feedback in the form of rewards or penalties, and learns over time to optimize actions based on a high Q-function. The authors utilized the DDQN for the optimization of parameters such as the base materials and thickness of the nanodisks and thin films used in the metasurface to create a hologram structure that operates in the visible spectrum and is independent of polarization. The DDQN agent initialized the parameters with initial values at the start of the optimization and iteratively refined the Q-function through trial and error. Through this process, we found the optimal result in just 2169 steps among approximately 5.7 billion possible states, and we were able to identify a structure with approximately twice the efficiency compared to the efficiency of existing structures with similar characteristics [77].

These examples demonstrate the feasibility of machine learning as an effective tool for the inverse calculation of the PnP. However, machine learning-based inverse design requires a substantial amount of initial data, incurring significant time and cost to compute the optimal topology using PnP. Therefore, obtaining a considerable amount of sample data through existing optimization methods should precede the effective application of machine learning to the PnP inverse design [12], [73].

## 6 Summary and outlook

In this review, we summarize the recent advancements in inverse design methods applied to proximity-field nanopatterning (PnP) to broaden the range of 3D nanostructures. Despite the versatility of PnPs in obtaining high pattern resolution and large fabrication areas, limitations persist in generating the desired 3D nanostructure topologies using conventional simulation and optimization methodologies. Hence, research aimed at increasing the design flexibility of 3D nanostructures using diverse inverse design tools is crucial.

First, they tailored lattices and motifs of the 2D design spaces by utilizing the adjoint method. Nontrivial lattices and motifs (e.g., a rectangular lattice with an elliptical motif), which could not be obtained using conventional methodologies, were achieved by introducing the adjoint method into the inverse design of PnP. Furthermore, the improved PSO algorithm can be applied to the design of phase masks for high-contrast nanostructures. The PSO algorithm is suitable for searching for global optima in non-differentiable and complex design fields because it does not use gradient values. They examined all parameters that determine the diffraction efficiencies of the phase mask and established the figure of merit (FoM) as an effective function to derive optimized phase mask geometries for achieving high contrast in electromagnetic waves at designated regions. Consequently, unconventional phase mask geometries can be obtained, leading to the creation of high-contrast nanostructures. Finally, we discuss recent achievements in machine-learning-based inverse design for phase modulation and its potential application to PnP.

However, there are still limitations that need to be addressed. Specifically, challenges remain in the production of arbitrary 3D nanotopologies using PnPs. Unlike the inverse design in 2D space using common simulation solvers, such as the finite difference time domain (FDTD), calculations in 3D space require significantly high computing power and data because their dimensionality increases. Optimizing simulation solvers for efficient calculations in 3D design space and preoccupying a large amount of initial data for deep learning are crucial research directions for realizing arbitrary patterns in 3D space. The application of solution for the inverse problem in scattering theory to the PnP inverse design also provides significant feasibility. The inverse problem of scattering theory involves obtaining the phase topology from a known scattered field, sharing similarities with PnP inverse design. Recently, Lee et al. focused on developing a converging Born series as an inverse problem solver [78]. Converging Born series demonstrated more than 500 times improved accuracy compared to FDTD and a computational speed 65 times faster, showing applicability for effective inverse problem solver. Finally, realizing a complex 3D topology using a single grating mask is challenging. Therefore, research efforts should focus on diversifying the range of phase-mask designs, including multilevel masks and phase masks that modulate the refractive index [52], [79]. One effective strategy to tailor the refractive index of the phase mask involves the use of composites. A recent study by Rho et al. demonstrated the rational design of the refractive index of a dielectric metalens by blending a photopolymeric resin with metal oxide nanoparticles possessing high refractive indices such as TiO_2_ and ZrO_2_ [79], [80]. They confirmed a linear control of the refractive index of the metalens based on the weight percentage of nanoparticles. Particularly, ZrO_2_ nanoparticles may be suitable as mask materials due to their higher refractive index (*n* ∼ 1.9) compared to PDMS (*n* ∼ 1.4) and low UV absorption at 355 nm. Research aimed at controlling the refractive index of the mask in this manner presents a promising avenue for further investigation in the field of PnP optimization. By gaining a comprehensive understanding of algorithmic technologies and their application in PnP, we anticipate establishing an essential pathway for the efficient production of 3D nanostructures with unprecedented levels of resolution and design flexibility.
